# A Case of Ligature and Division of the Vasa Deferentia for Prostatic Hypertrophy

**Published:** 1896-06

**Authors:** C. W. J. Brasher


### A CASE OF LIGATURE AND DIVISION OF THE VASA DEFERENTIA FOR PROSTATIC HYPERTROPHY.

BY

C. W. J. Brasher, M.R.C.S. Eng., L.R.C.P. Lond.

---

The operations of ligature or of division of the vasa deferentia in cases of hypertrophy of the prostate, have been recently recommended as alternatives to the more heroic procedure of castration, amongst others by Ewing Mears[1] of Philadelphia, and by Pavone of Turin.[2] These operations have been markedly successful in the hands of Isnardi of Turin,[3] and of Tilden Brown of New York.[4] Mr. Reginald Harrison also mentions[5] a case in which he performed subcutaneous division of the vasa deferentia with a successful result, and, more recently, he has recorded three successful cases of ligature of the vas.

Whilst castration, double or single, has yielded most remarkable results—a considerable percentage of the patients being able to discard the catheter entirely, and a great number of the other patients experiencing great relief—several fatal cases have shown that it is occasionally attended by considerable danger; and this fact, apart from the natural objection most patients feel to an operation involving an obvious mutilation, is sufficient to justify a more extended trial of a comparatively trifling operation, and one which would not preclude subsequent castration as a last resource.

In the case which I am about to relate, the two operations were suggested to the patient, and it was pointed out that

[1] *Ann. Surg.*, 1895, xxi. 181.

[2] *Policlin.*, 1895, ii. 225; abstract in *Brit. M. J.*, 1895, ii., Epitome, p. 2.

[3] *Centralbl. f. Chir.*, 1895, xxii. 657; abstract in *Brit. M. J.*, 1895, ii., Epitome, p. 30. *Riforma med.*, June 19, 1895; abstract in *Brit. M. J.*, 1895, ii., Epitome, p. 42.

[4] *J. Cutan. & Genito-Urin. Dis.*, 1896, xiv. 21.

[5] *Brit. M. J*, 1895, ii. 1605.

castration might be relied upon to relieve the urgent symptoms, whilst ligature of the vas deferens was less certain in its results. The patient was obviously disconcerted at the prospect of "losing his manhood," as he termed it, and consented eagerly to the alternative operation.

Mr. R. J., aged 79, has always been a very nervous, excitable individual, and for many years was a heavy drinker. He has always been healthy until five years ago, when he had a sudden attack of retention of urine, lasting thirty-six hours. His medical attendant passed a catheter *à boule*, and instructed the patient in its use. Since that time he has found it necessary to use the instrument more and more frequently, until, for a month before I saw him, he had been unable to pass any urine naturally, and he had observed that the difficulty in passing the catheter had steadily increased.

The patient sent for me on Monday, December 15th, 1895, as he had been unable to pass the catheter since the previous Saturday. I found him walking about his room in great pain, and he showed me four broken catheters, the result of his ineffectual efforts to relieve himself. With some difficulty I succeeded in passing a black *coudée* catheter with a stilette, and drew off about 2¼ pints of urine, which was of a port-wine colour from the quantity of blood it contained. This urine was neutral in reaction; sp. gr. 1022; no sugar. There was some urethritis with great spasm of the urethral muscles, almost, in fact, amounting to a spasmodic stricture, but there was no organic stricture. *Per rectum*, the prostate was found to be enormously enlarged, apparently to the size of a Tangerine orange. The tip of the index-finger could not be passed beyond it, and it felt almost as hard as a solid mass of india-rubber.

It now became necessary to pass the catheter three or four times a day, and the resistance was so great that the stilette was always needed. The prostate bled very freely every time the catheter was used, and there was no diminution in its size after rest in bed and routine treatment continued for several days. The patient, alarmed by the frequent loss of blood, was most anxious for an operation; but, as I have mentioned, shrank from submitting to castration. It was therefore decided to ligature and divide the vasa deferentia.

It occurred to me that if the atrophy of the prostate following these operations were secondary to the atrophy of the testes, this result might be brought about more quickly, and at the same time the congestion of the prostatic plexus would be somewhat relieved, if the spermatic arteries were ligatured during the operation.

On December 24th, with the assistance of Mr. Freeman and Mr. Rayner, I divided both vasa deferentia between double catgut ligatures, and excised about $\frac{1}{4}$ inch of the vas on the left side. The spermatic arteries were also ligatured. The right one was easily recognised by its pulsation towards the anterior part of the cord, but the left one was much more difficult to find on account of there being a varicocele on that side. There was also a hydrocele on the left side, which was emptied with the exploring syringe before the operation. The wounds healed by first intention, and the patient made fairly rapid progress.

The third time the catheter was passed after the operation the urine was quite free from blood, and was tested for albumen with a negative result. The reaction was slightly acid, and has been acid ever since.

The day after the operation (December 25th) 6 oz. of urine were passed naturally. I regarded his as a coincidence, but on the following day 9 oz. were pas d, and on the third day after the operation the quantity passed naturally was 14 oz. The urethritis and urethral spasm persisted for many weeks and retarded the patient's recovery; and he has had, unfortunately, three attacks of bronchitis (accompanied by much vesical irritation and catarrh) since the operation, and during these attacks the retention returned to some extent. He has, however, been able to go repeatedly for two days without requiring the catheter, and although, after the last attack of bronchitis, it was passed every night for some weeks, the average amount of residual urine was only 5 oz. or 6 oz.—about $\frac{1}{5}$ of the total quantity evacuated in twenty-four hours.

The patient now finds that his bladder will hold 8 oz. or 10 oz. of urine without much discomfort, but he cannot pass more than 2 oz. or $2\frac{1}{2}$ oz. as a rule before the sphincter of the bladder and the urethral muscles contract spasmodically, and he has to wait a few minutes before he can pass any more. When he first began to micturate naturally after the operation, the quantity evacuated on each occasion was never more than $\frac{1}{2}$ oz., so there has been steady improvement in that respect. He has also observed that when a larger quantity of urine than usual is secreted during the night (*e.g.* 15 oz. or 20 oz.), and the bladder becomes distended, the first attempt at micturition brings on a powerful contraction of the sphincter, which can only be overcome by the catheter; but these attacks are becoming less frequent.

Since the beginning of April (sixteen weeks after the operation), with two exceptions, the patient has only required the catheter to be used on alternate nights, and, once or twice, every third night has been sufficient. The quantity of residual urine is from 5 oz. to 7 oz. He has improved greatly in general health, and has gained flesh; he is very seldom obliged to get up to micturate before 6 or 7 a.m., and he finds every week that he has to strain less during the act.

The prostate has slowly decreased in size, until now (April 24) it appears to be somewhat less than two-thirds its former size, the finger passing well beyond it, and it is *flatter* than when first examined.

For about three months after the operation the testes were markedly smaller and very tender on pressure, whilst there was much "matting" and thickening of the cords, chiefly on the proximal side of the wound. The testes have now nearly regained their former size, the tenderness has disappeared, and the thickening of the spermatic cords can hardly be felt. The right epididymis is large, and, as has been observed by Isnardi, decidedly nodular. The varicocele on the left side cannot be felt; but the hydrocele has filled up to some extent, although it is not yet necessary to empty it.

Although my patient's progress has not been so rapid as that of some of the patients whose cases have been recorded, he is still improving. Bearing in mind his advanced age, the great hardness of the prostate, and the long duration of difficult micturition, I think it is doubtful whether he would have improved more rapidly if castration had been done; whilst his nervous and excitable temperament and great repugnance to that operation must have increased its danger.

Several surgeons who have had considerable experience in these operations consider that the ligature of the spermatic arteries is unnecessary; but when, as in the present case, the patient is losing a large quantity of blood every time the catheter is passed, and it is necessary to lower the vitality of the testes as quickly as possible, the ligature of the artery may help to bring about that result more quickly than if the vas deferens only were divided.

Table showing the total quantities of urine passed naturally, and drawn off by catheter, from December 24th (operation) to April 24th (17½ weeks):

| | Quantity passed naturally. oz. | Residual urine. oz. |
|---|---|---|
| Dec. 24th — 31st | 77 | 200 |
| Jan. 1st — 7th | ? [1] | 59 |
| ,, 8th — 14th | 78½ [2] | 128 |
| ,, 15th — 21st | 146½ | 44 |
| ,, 22nd — 28th | 113½ | 26½ |
| ,, 29th—Feb. 4th | 131½ | 20½ |
| Feb. 5th — 11th | 106½ [2] | 100½ |
| ,, 12th — 18th | 33½ | 115 |
| ,, 19th — 25th | 97 | 70 |
| ,, 26th—Mar. 3rd | 44 [2] | 159 |
| Mar. 4th — 10th | 132 | 68 |
| ,, 11th — 17th | 109½ | 89 |
| ,, 18th — 24th | 144½ | 39½ |
| ,, 25th — 31st | 190 | 39 |
| April 1st — 7th | 177 | 22½ |
| ,, 8th — 14th | 170 [3, 4] | 43 |
| ,, 15th — 21st | 196½ [3, 5] | 48½ |
| ,, 22nd — 24th (3 days). | 90½ | 5 |

[1] Quantity could not be measured, patient had diarrhœa.
[2] Bronchitis and vesical catarrh.
[3] Retention occurred one morning this week from over-distension of bladder.
[4] Patient drew off 24 oz. in early morning (April 9).
[5] Patient drew off 15 oz. in early morning (April 17).

May 15th.—"The patient has made fairly rapid progress towards almost complete recovery, and at this date—20½ weeks after the operation, he has not required the catheter for a week, and only micturates ten or twelve times in twenty-four hours. His general health is so good that he can walk up some of the steepest hills in Bristol."

